# Complicated acute haematogenous osteomyelitis with fatal outcome following a closed clavicle fracture—a case report and literature review

**DOI:** 10.1259/bjrcr.20150389

**Published:** 2016-05-01

**Authors:** Tina Kocutar, Žiga Snoj, Vladka Salapura

**Affiliations:** Clinical Institute of Radiology, University Medical Centre Ljubljana, Ljubljana, Slovenia

## Abstract

Among adults, post-traumatic osteomyelitis following a closed fracture is a rarely described entity in the literature, with the involvement of the clavicle bone being particularly uncommon. Early diagnosis and treatment of clavicular osteomyelitis is crucial to prevent serious consequences such as sepsis, mediastinitis and haemorrhage from the great vessels. A 54-year-old male patient presented to the emergency department complaining of fatigue and limited mobility after having fallen and hit his head and right shoulder 10 days previously. No major injury was found during the diagnostic procedure, and the patient was discharged. 2 weeks later, the patient returned with clinical signs of right upper arm cellulitis and probable sepsis. Diagnostic ultrasound imaging and MRI of the right upper arm, as well as re-examination of the X-ray image, confirmed acute complex osteomyelitis of the right clavicle following an overlooked clavicle fracture. Microbiological analysis confirmed clavicular osteomyelitis caused by *Escherichia coli* septicaemia. Despite prompt treatment with i.v. antibiotics and surgery, the patient's condition rapidly deteriorated and he passed away. Our case demonstrates the critical importance of early diagnosis and appropriate treatment of a closed fracture. Late diagnosis may lead to severe complications, such as complicated osteomyelitis and sepsis, and even a fatal outcome. Furthermore, a brief literature review is presented of previously reported acute osteomyelitis following a closed fracture, including evidence of affected bone and isolated pathogens. Although uncommon, osteomyelitis should be considered a possible cause of a deteriorating clinical condition in patients with a history of recent trauma.

## Introduction

Osteomyelitis of the clavicle is an uncommon form of infection caused by haematogenous or contiguous spread, or trauma.^1–3^ This rare condition is difficult to diagnose. Pain and swelling of the medial end of the clavicle may be associated with osteoarthritis, infection or condensing osteitis, and it can be difficult to differentiate between these diagnoses from clinical and radiological perspectives.^3^ Despite modern surgical techniques and advances made in antimicrobial therapy, osteomyelitis remains a difficult and challenging problem.^3–5^

Acute osteomyelitis caused by haematogenous spread predominantly affects children because their metaphyseal regions are highly vascular and susceptible to even minor trauma. Over half of the cases of acute haematogenous osteomyelitis in children occur in patients younger than 5 years of age.^6^ Haematogenous osteomyelitis among adults is most commonly seen in immunosuppressed patients and patients with underlying medical conditions (*e.g.* diabetes mellitus, cancer, chronic renal disease) or a history of i.v. drug use.^7–9^ In adults, osteomyelitis typically involves the vertebrae, but can occur in the long bones, pelvis or clavicle.^10^ The common presentation of osteomyelitis includes the insidious onset of a dull pain, with or without signs of fever, tenderness, swelling and erythema. The formation of a soft tissue abscess, fluctuation or discharge appears late in the course of the disease.^9^

Post-traumatic osteomyelitis can occur in up to 26% of open fractures.^11^ However, acute haematogenous osteomyelitis following a closed fracture is an uncommon complication in adults, with the involvement of the clavicle being exceptionally rare.^1,12^ Reports in the literature agree that aggressive and early treatment is strongly indicated.^13–15^ The aim of the treatment for clavicular osteomyelitis is to eliminate the infection in order to prevent serious consequences such as sepsis, mediastinitis and haemorrhage from the great vessels. Intravenous antibiotics should be instituted as soon as the diagnosis is suspected, and continued for 4–8 weeks. Wide local debridement represents the mainstay of the treatment.^16^ The presented case describes a unique example of acute osteomyelitis with rapid progression, serious complications and a fatal outcome after an overlooked closed fracture of the clavicle. Furthermore, a brief literature review is presented of previously reported papers of acute osteomyelitis following a closed fracture.

## Case report

**Figure 1.**
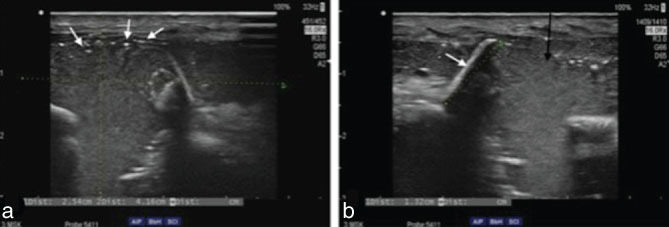
Ultrasonography of the supraclavicular region. (a) Subcutaneous hyperechoic, complex fluid collection extending above the right clavicle and between the clavicle fragments, with the presence of hyperechoic gas bubbles (white arrows). (b) A free fragment of the clavicle cortical bone (white arrow) surrounded with thick, hyperechoic fluid collection (black arrow).

**Figure 2.**
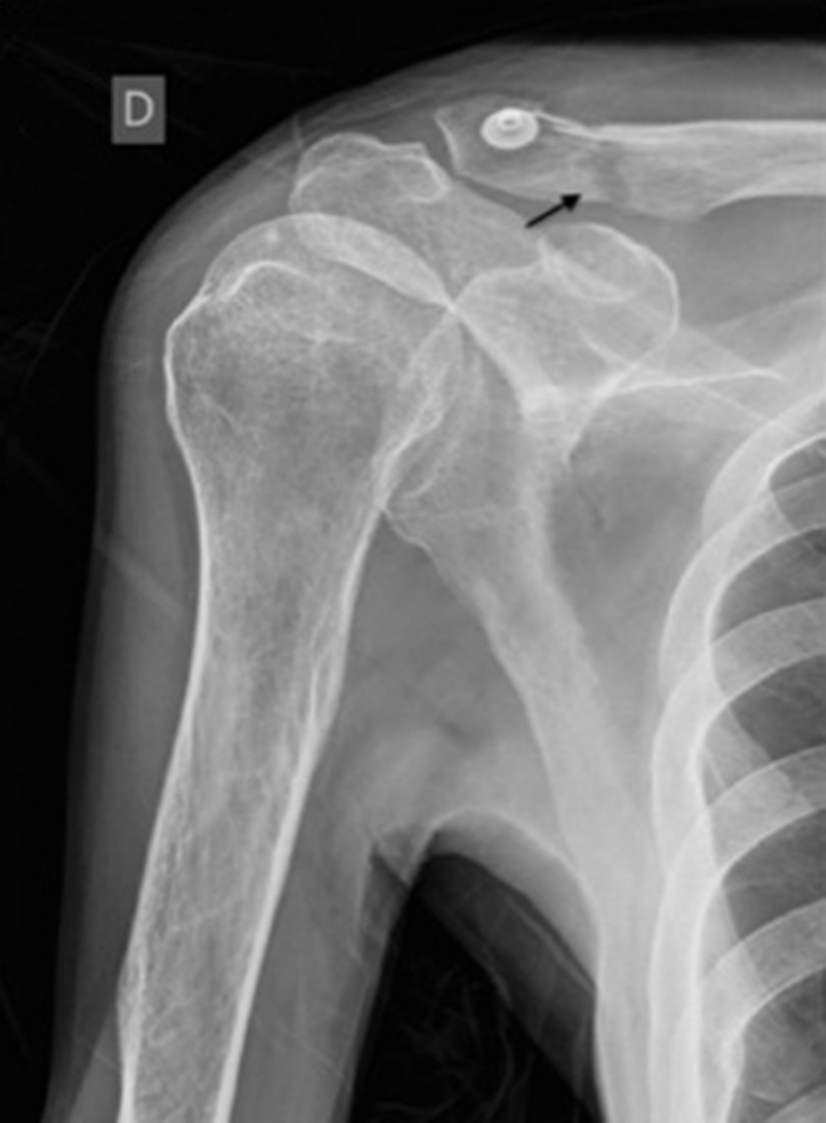
X-ray image of the right shoulder. A wide, lucent fracture line without dislocation between the fragments is seen at the distal third of the right clavicle (black arrow). The X-ray was taken during the patient’s first visit to the emergency department and was misinterpreted as normal.

**Figure 3.**
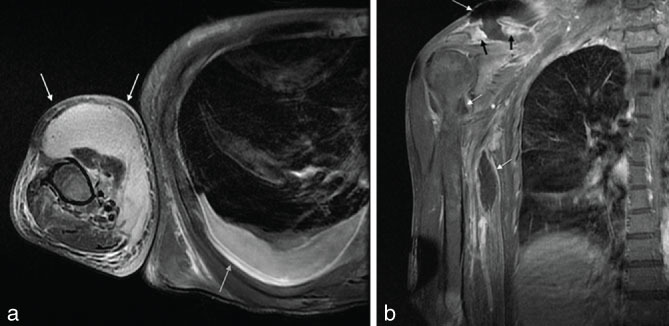
MRI of the right shoulder. (a) *T*_2_ fast relaxation fast spin-echo fat-saturated sequence, axial images. Continuation of a large abscess distally encompassing the anterior compartment of the right arm. The fluid collection extended almost to the level of the elbow (white arrows). An additional finding was the moderate right pleural effusion (grey arrow). (b) *T*_1_ fast spin-echo fat-saturated post-contrast images in the coronal plane. Clavicle fracture and destruction with a markedly diffuse, inflammatory post-contrast enhancement of the clavicle (black arrows). Peripheral ring enhancement of the supraclavicular and brachial abscess formation is evident (white arrows).

A 54-year-old male patient presented to the emergency department complaining of fatigue and increasingly limited mobility in his right arm. According to his records, the patient had fallen 10 days previously and hit his head and right shoulder. Right-sided subcutaneous facial haematoma and a contusion mark on the right shoulder were observed during physical examination. The examination showed a moderately limited range of motion in the right shoulder. A CT scan of the head and an X-ray of the cervical spine and right shoulder were performed. The CT scan of the head and the X-ray of the cervical spine showed no signs of injury, and the X-ray of the right shoulder was interpreted as normal. Besides a history of smoking and excessive drinking, the medical history was unremarkable. Laboratory examination showed a slightly elevated C-reactive protein inflammatory marker (22 mg l^–1^), anaemia (red blood cell count = 3.02 × 10^12^ l^–1^, haemoglobin = 105 g l^–1^), thrombocytopenia (platelet count = 46 × 10^9^ l^–1^), low haematocrit (31%) and an alcohol blood concentration of 55 mmol l^–1^. As no major injury was found, the patient was discharged. 2 weeks later, the patient returned to the emergency department complaining of fatigue, diffuse arthralgia and myalgia, with severe pain in his right shoulder. The skin on his right upper arm was swollen, reddened and painful to palpation, and his right axillary lymph nodes were enlarged. On examination, the patient had a pulse of 92 beats min^–1^, blood pressure of 68/38 mmHg, oxygen saturation of 97% and a temperature of 36°C. Laboratory examination showed an elevated C-reactive protein of 129 mg l^–1^, a white cell count of 11.6 × 10^9 ^l^–1^, red blood cell count of 2.09 × 10^12^ l^–1^, haemoglobin of 70 g l^–1^, a haematocrit of 21% and a gamma glutamyl transferase of 1.36 μkat l^–1^. The patient was hospitalized and diagnosed with right upper arm cellulitis and probable sepsis. An emergency ultrasound examination of the upper arm showed a collection of thick fluid with the presence of gas bubbles and a free fragment of the cortical bone (Figure 1). At this point, the shoulder X-ray image that was taken during the patient’s first visit to the hospital was re-examined, and a clavicle fracture in the distal third of the clavicle was diagnosed (Figure 2). In order to assess the anatomical relations between the collection of fluid and its adjacent structures and to better evaluate the extent of bone involvement, we immediately performed an MRI on a Signa scanner 1.5 T (General Electric Medical Systems, Milwaukee, WI). The imaging protocol was performed using a short tau inversion–recovery sequence (coronal plane), a *T*_2 _fast relaxation fast spin-echo (FSE) fat-saturated sequence (axial plane) and a *T*_1_ FSE sequence (coronal plane) before i.v. application of the paramagnetic contrast media, and afterwards with a *T*_1_ FSE fat-saturated sequence (coronal, axial plane). The MRI confirmed osteomyelitis of the clavicle and moderate right-sided pleural effusion (Figure 3a,b). Apart from these MRI findings, the X-ray of the lungs also showed radiological signs of possible infiltration of the right lower lobe.

Empirical therapy began with 2 g 6 h^–1^ of i.v. floxacillin and, after *Streptococcus pneumoniae* and *E. coli* grew in the blood cultures, 2 g 6 h^–1^ of i.v. cefotaxime was added to the therapy. No bacteria were isolated from the right pleural punctuate and uroculture. A thorough physical examination revealed a deep 1 × 2 cm wound on the patient’s left fourth toe, exposing the underlying tendon. The wound showed no signs of infection, but polybacterial flora grew on the smear taken from the wound, in which the presence of *E.coli* was identified. The patient was not a suitable candidate for immediate operation owing to abnormal haemostasis (prothrombin time = 68.9 s; international normalized ratio= 2.45) and profound anaemia (treated with supportive therapy with three ampoules of phytomenadione intramuscular and six units of concentrated thrombocytes i.v.), until 2 days later, at which point surgical debridement and drainage were performed. A day prior to the surgery, there was a spontaneous discharge from the clavicle area of approximately 500 ml of pus. *E. coli* grew on a smear of the intraoperative right clavicular wound and a clavicle tissue sample.

Despite surgical treatment, the patient’s condition deteriorated rapidly during the next 48 h with the development of acute respiratory distress syndrome and cardiac arrest with asystole. After successful cardiopulmonary resuscitation and the return of spontaneous circulation, systemic inflammatory response syndrome developed with multiple organ failure. The patient’s condition deteriorated rapidly, and he passed away the following day.

## Discussion

The case presented demonstrates the critical importance of early diagnosis of acute osteomyelitis and the appropriate treatment of a closed fracture, as well as the importance of preventive treatment of skin and soft tissue wounds, which present a potential risk for local and systemic bacterial infection. This is especially important in patients with chronic diseases or otherwise immunocompromised patients, who are at greater risk of developing these complications.^17,18^ The patient in the case presented was diagnosed with advanced osteomyelitis, as a closed clavicle fracture had previously been overlooked, resulting in no standard therapy and follow-up. A chronic deep skin wound also had not been previously aetiologically diagnosed or treated, which could have presented an important preventative measure against later complications. Furthermore, this case demonstrates that osteomyelitis, although a very uncommon complication, should be considered a possible cause of a deteriorating clinical condition in patients with a history of recent trauma. All of these factors are crucial in contributing to an adequate treatment outcome.

With very few reported cases in the literature, haematogenous osteomyelitis following a closed bone fracture is an uncommon entity among adults. A MEDLINE search revealed that there have been 18 cases of osteomyelitis following a closed bone fracture in adults since 1976.^19–33^ Waldvogel et al^34^ described the involvement of the clavicle in an infective process as being uncommon and usually secondary to the spread of infection from adjacent areas, and is often associated with predisposing factors such as head and neck surgery, radiation therapy, subclavian vein catheterization or immunosuppression in transplant patients. Concomitant with this statement, different authors have reported rare cases of clavicular osteomyelitis following central line placement,^35^ Swan–Ganz catheterization,^36^ trauma^16^ and as a complication after head and neck surgery.^3,16^ Our brief literature review revealed that *Staphylococcus aureus* appears to be the most commonly isolated pathogen in reported cases of haematogenous osteomyelitis following closed fractures.^37^ Other commonly isolated pathogens are *Staphylococcus epidermidis, Pseudomonas aeruginosa, Serratia marcescens* and *E. coli*.^38^

To our knowledge, only one case of acute haematogenous osteomyelitis following a closed fracture of the clavicle in an adult has been reported. The patient had Crohn’s disease and previous sternotomies, and had developed *Salmonella* sternoclavicular osteomyelitis subsequent to *Salmonella* enteritidis sepsis.^33^

The patient in our case suffered from a closed clavicle fracture without dislocation, which had been overlooked during his first visit to the emergency department (Figure 2). The upper arm of the patient was not immobilized when he was discharged from the hospital. Acute osteomyelitis of the right clavicle with severe complications subsequently developed. *S. pneumoniae* and *E. coli* grew in the blood cultures, and a clavicle tissue sample taken with a wound smear during surgery proved *E. coli* to be the cause of the haematogenous osteomyelitis. Isolation of bacteria from a bone biopsy sample, together with histologic findings of inflammation and osteonecrosis, is the reference standard for a diagnosis of osteomyelitis.^39^

In terms of the underlying conditions that could have contributed to this devastating clinical course of haematogenous osteomyelitis in the patient, there are several factors that can be considered. One very possible factor is the immunocompetence of the patient, which may have been impaired owing to his long history of chronic alcoholism. *E. coli* has been described in the literature as a very commonly isolated Gram-negative bacilli in septicaemia.^40^ *E. coli* and *S. pneumoniae* are more commonly isolated pathogens in cases of septicaemia in chronic alcoholism than in the general population.^41^ The most common foci of *E. coli* septicaemia are urinary tract infections; however, Gram-negative bacilli in acutely ill, debilitated patients, alcoholics; diabetics,and those with chronic bronchitis may also lead to *E. coli* pneumonia.^40,42^ Besides profound anaemia, concomitant *S. pneumoniae* septicaemia and right lower lobe pneumonia suggest the poor immune status of the patient in this study. In search of a probable primary focus of *E. coli* septicaemia, several different smears were taken, and *E. coli* was isolated from a deep skin wound on the patient’s left fourth toe. All other tests were negative (pleural effusion sample, Sanford urine test). This finding underlines the importance of the preventive treatment of skin wounds in immunocompromised patients, especially in specific body areas with a greater microbial burden.^17^ The prevention of infection demands gentle care of the wound in order to prevent additional trauma, maceration or alteration of normal microbial flora. If the wound becomes infected, it is vital that it is aetiologically diagnosed and specifically treated.^18^ For the patient in this case, the chronic wound on his foot had previously been untreated. Second, the patient’s fracture had not been treated in the usual manner, that is, through immobilization with a figure-of-eight bandage or a broad arm sling,^43^ as it was only diagnosed afterwards. It can also be argued that these factors worked in synergy or that other unidentified factors contributed to the rapid development of the disease.

A diagnosis of osteomyelitis in adults can be challenging and requires a high index of clinical suspicion.^7^ Imaging techniques play a key role in the early diagnosis and follow-up of trauma. In the event that osteomyelitis does develop, early diagnosis is critical, as prompt antibiotic therapy and surgical intervention may prevent necrosis of the bone. An inadequate or late diagnosis significantly diminishes the cure rate and increases the degree of complications and morbidity.^44^ In our case, although osteomyelitis was diagnosed as soon as the patient presented to the hospital for the second time, the disease was already in an advanced state. Despite prompt treatment with antibiotics and surgery, the infection developed rapidly and aggressively, and the patient’s condition deteriorated.

## Conclusions

Early diagnosis of acute osteomyelitis and appropriate treatment of closed fractures are vital for a successful treatment outcome. Early diagnosis is crucial, since late diagnosis may lead to severe complications, such as complicated osteomyelitis and sepsis, and even a fatal outcome. This is especially important in patients with chronic diseases or otherwise immunocompromised status. Although uncommon, osteomyelitis should be considered a possible cause of a deteriorating clinical condition in patients with a history of recent trauma.

## Learning points

1. Osteomyelitis of the clavicle is an uncommon form of infection caused by haematogenous or contiguous spread, or trauma.
2. Acute osteomyelitis caused by haematogenous spread predominantly affects children. Haematogenous osteomyelitis in adults is most often seen in patients with immunosuppressed status or with underlying medical conditions or a history of i.v. drug use.
3. Acute haematogenous osteomyelitis following a closed fracture is an uncommon complication in adults, with the involvement of the clavicle being especially rare.
4. Early diagnosis of osteomyelitis is critical, since prompt antibiotic therapy and surgical intervention may prevent necrosis of the bone.
5. Preventive treatment of skin and soft tissue wounds, as a potential focus of bacterial superinfection, which may lead to systemic infection with further complications, is especially important in immunocompromised patients.

## Consent

We were unable to obtain a signed informed consent form from the patient or his next of kin; however, the National Medical Ethics Committee acknowledges that exhaustive attempts have been made to contact the patient’s next of kin, to no avail, and that all information with imaging material has been sufficiently anonymized.

## References

[1] Waldvogel FA, Medoff G, Swartz MN Osteomyelitis: a review of clinical features, therapeutic considerations and unusual aspects. N Engl J Med 1970; 282: 198–206. 4902833 10.1056/NEJM197001222820406

[2] Sonobe M, Miyazaki M, Nakagawa M, Ikegami N, Suzumura Y, Nagasawa M, et al Descending necrotizing mediastinitis with sternocostoclavicular osteomyelitis and partial thoracic empyema: report of a case. Surg Today 1999; 29: 1287–9. 10639715 10.1007/BF02482226

[3] Balakrishnan C, Vashi C, Jackson O, Hess J Post-traumatic osteomyelitis of the clavicle: a case report and review of literature. Can J Plast Surg 2008; 16: 89–91. 19554172 10.1177/229255030801600208 PMC2691560

[4] Cremieux AC, Carbon C Experimental models of bone and prosthetic joint infections. Clin Infect Dis 1997; 25: 1295–302. 9431367 10.1086/516135

[5] Norden CW Bone and joint infection. Curr Opin Infect Dis 1996; 9: 109–14.

[6] Gutierrez K Bone and joint infections in children. Pediatr Clin North Am 2005; 52: 779–94. 15925662 10.1016/j.pcl.2005.02.005

[7] Hatzenbuehler J, Pulling TJ Diagnosis and management of osteomyelitis. Am Fam Physician 2011; 84: 1027–33. 22046943

[8] Zaha H, Onomura M, Nishikuramori Y Pyogenic vertebral osteomyelitis in a breast cancer patient: report of a case. Surg Today 2012; 42: 1022–5. 22382854 10.1007/s00595-012-0158-0

[9] Thein R, Tenenbaum S, Chechick O, Leshem E, Chechik A, Liberman B Delay in diagnosis of femoral hematogenous osteomyelitis in adults: an elusive disease with poor outcome. Isr Med Assoc J 2013; 15: 85–8. 23516768

[10] Zimmerli W Vertebral osteomyelitis. N Engl J Med 2010; 362: 1022–9. 20237348 10.1056/NEJMcp0910753

[11] Merritt K Factors increasing the risk of infection in patients with open fractures. J Trauma 1988; 28: 823–7. 3385826 10.1097/00005373-198806000-00018

[12] Gerscovich E, Greenspan A Osteomyelitis of the clavicle: clinical, radiologic, and bacteriologic findings in ten patients. Skeletal Radiol 1994; 23: 205–10. 8016673 10.1007/BF00197463

[13] Granick MS, Ramasastry SS, Goodman MA, Hardesty R Chronic osteomyelitis of the clavicle. Plast Reconstr Surg 1989; 84: 80–4. 2734407 10.1097/00006534-198907000-00015

[14] Alessi DM, Sercarz JA, Calcaterra TC Osteomyelitis of the clavicle. Arch Otolaryngol Head Neck Surg 1988; 114: 1000–2. 3408564 10.1001/archotol.1988.01860210066017

[15] Baratz M, Appleby D, Fu FH Life-threatening clavicular osteomyelitis in two debilitated patients. Orthopedics 1985; 8: 1492–4. 3832035 10.3928/0147-7447-19851201-09

[16] Burns P, Sheahan P, Doody J, Kinsella J, Rosenthal EL Clavicular osteomyelitis: a rare complication of head and neck cancer surgery. Head Neck 2008; 30: 1124–7. 18228522 10.1002/hed.20762

[17] Dissemond J, Assadian O, Gerber V, Kingsley A, Kramer A, Leaper DJ, et al Classification of wounds at risk and their antimicrobial treatment with polihexanide: a practice-oriented expert recommendation. Skin Pharmacol Physiol 2011; 24: 245–55. 21508658 10.1159/000327210

[18] Stevens DL, Bisno AL, Chambers HF, Everett ED, Dellinger P, Goldstein EJ, et al Infectious Diseases Society of America Practice guidelines for the diagnosis and management of skin and soft-tissue infections. Clin Infect Dis 2005; 41: 1373–406. 16231249 10.1086/497143

[19] Aluisio FV, Scully SP Acute hematogenous osteomyelitis of a closed fracture with chronic superinfection. Clin Orthop Relat Res 1996; 325: 239–44. 10.1097/00003086-199604000-00029 8998882

[20] Ramos Martínez A, Duca A, Muñez Rubio E, Valverde Herreros ML, Ramírez Feito C Osteomyelitis due to Escherichia coli complicating a closed humeral fracture. An Med Interna 2006; 23: 588–90. 17371148 10.4321/s0212-71992006001200008

[21] Watson FM, Whitesides TE Acute hematogenous osteomyelitis complicating closed fractures. Clin Orthop Relat Res 1976; 117: 296–302. 1277679

[22] Enat R, Pollack S, Wiener M, Barzilai D Osteomyelitis in fractured sternum after cardiopulmonary resuscitation. N Engl J Med 1979; 301: 108–9. 10.1056/nejm197907123010217 449937

[23] Mensah GA, Gold JP, Schreiber T, Isom OW Acute purulent mediastinitis and sternal osteomyelitis after closed chest cardiopulmonary resuscitation: a case report and review of the literature. Ann Thorac Surg 1988; 46: 353–5. 3046524 10.1016/s0003-4975(10)65946-2

[24] Mallinson RH, Tremlett CH, Payne BV, Richards JE Sternal osteomyelitis after cardiopulmonary resuscitation. J R Soc Med 1999; 92: 87. 10450222 10.1177/014107689909200213 PMC1297070

[25] Randell PA, Somers L Case of the month: "bugs are eating my soul" - sternal abscess, osteomyelitis, and mediastinitis complicating a closed sternal fracture. Emerg Med J 2006; 23: 736–7. 16921099 10.1136/emj.2006.038000 PMC2564229

[26] Ahmar W, Morley P, Marasco S, Chan W, Aggarwal A Sternal fracture and osteomyelitis: an unusual complication of a precordial thump. Resuscitation 2007; 75: 540–2. 17697738 10.1016/j.resuscitation.2007.05.017

[27] Ebong WW Acute osteomyelitis three years after a closed fracture in an adult with sickle-cell anemia. A case report. J Bone Joint Surg Am 1980; 62: 1196–8. 7430209

[28] Abrahams MA, Tylkowski CM Brucella osteomyelitis of a closed femur fracture. Clin Orthop Relat Res 1985; 195: 194–6. 3919984

[29] Baskaran S, Nahulan T, Kumar AS Close fracture complicated by acute haematogenous osteomyelitis. Med J Malaysia 2004; 59 Suppl F: 72–4. 15941170

[30] Govender S, Charles RW, Ballaram RS, Achary DM Vertebral osteomyelitis after a closed fracture of the spine. A case report. S Afr Med J 1988; 73: 124–6. 3340917

[31] Fellmeth BD, DaSilva RM, Spengler DM Hematogenous osteomyelitis complicating a closed compression fracture of the spine. J Spinal Disord 1988; 1: 168–71. 2980074

[32] Fox IM, Brady K Acute hematogenous osteomyelitis in intravenous drug users. J Foot Ankle Surg 1997; 36: 301–5. 9298447 10.1016/s1067-2516(97)80077-4

[33] Tickell KD, Banim R, Kustos I Salmonella sternoclavicular osteomyelitis in a patient with crohn's disease. BMJ Case Rep 2013; 2013. 10.1136/bcr-2012-007809 PMC3603608 23345484

[34] Waldvogel FA, Vasey H Osteomyelitis: the past decade. N Engl J Med 1980; 303: 360–70. 6993944 10.1056/NEJM198008143030703

[35] Manny J, Haruzi I, Yosipovitch Z Osteomyelitis of the clavicle following subclavian vein catheterization. Arch Surg 1973; 106: 342–3. 4689809 10.1001/archsurg.1973.01350150076020

[36] Hunter D Osteomyelitis of the clavicle after Swan-Ganz catheterization. Arch Intern Med 1983; 143: 153–4. 6849595

[37] Cullen JR, Primrose WJ, Vaughn CW Osteomyelitis as a complication of a tracheo-oesophageal puncture. J Laryngol Otol 1993; 107: 242–4. 8509706 10.1017/s0022215100122753

[38] Carek PJ, Dickerson LM, Sack JL Diagnosis and management of osteomyelitis. Am Fam Physician 2001; 63: 2413–20. 11430456

[39] Lew DP, Waldvogel FA Osteomyelitis. Lancet 2004; 364: 369–79. 15276398 10.1016/S0140-6736(04)16727-5

[40] Eykyn SJ, Gransden WR, Phillips I The causative organisms of septicaemia and their epidemiology. J Antimicrob Chemother 1990; 25: 41–58. 2189856 10.1093/jac/25.suppl_c.41

[41] Siboni A Serious infections in alcoholics. 2. Etiology of bacteremia and meningitis in alcoholics discharged from hospitals in Funen 1981, 1984 and 1986. Ugeskr Laeger 1989; 151: 376–81. 2919457

[42] Levison ME, Kaye D Pneumonia caused by gram-negative bacilli: an overview. Clin Infect Dis 1985; 7: S656–S665. 10.1093/clinids/7.supplement_4.s656 3909320

[43] Stanley D, Norris SH Recovery following fractures of the clavicle treated conservatively. Injury 1988; 19: 162–4. 3248891 10.1016/0020-1383(88)90006-x

[44] Pineda C, Espinosa R, Pena A Radiographic imaging in osteomyelitis: the role of plain radiography, computed tomography, ultrasonography, magnetic resonance imaging, and scintigraphy. Semin Plast Surg 2009; 23: 80–9. 20567730 10.1055/s-0029-1214160 PMC2884903

